# Insight into Potential Interactions of Thyroid Hormones, Sex Hormones and Their Stimulating Hormones in the Development of Non-Alcoholic Fatty Liver Disease

**DOI:** 10.3390/metabo12080718

**Published:** 2022-08-04

**Authors:** Eleonore Fröhlich, Richard Wahl

**Affiliations:** 1 Center for Medical Research, Medical University Graz, 8010 Graz, Austria; 2 Institute for Clinical Chemistry and Pathobiochemistry, Department for Diagnostic Laboratory Medicine, University Hospital Tuebingen, Hoppe-Seyler Str. 3, 72076 Tuebingen, Germany

**Keywords:** thyroid-stimulating hormone, follicle-stimulating hormone, metabolic syndrome, hypothyroidism, menopause, metabolic dysfunction-associated fatty liver disease

## Abstract

Non-Alcoholic Fatty Liver Disease (NAFLD) is a common manifestation of metabolic syndrome. In addition to lifestyle, endocrine hormones play a role in the dysregulation of hepatic metabolism. The most common endocrine hormones contributing to metabolic syndrome are alterations in the levels of thyroid hormones (THs, predominantly in subclinical hypothyroidism) and of sex hormones (in menopause). These hormonal changes influence hepatic lipid and glucose metabolism and may increase hepatic fat accumulation. This review compares the effects of sex hormones, THs and the respective stimulating hormones, Thyroid-Stimulating Hormone (TSH) and Follicle-Stimulating Hormone (FSH), on the development of hepatosteatosis. TSH and FSH may be more relevant to the dysregulation of hepatic metabolism than the peripheral hormones because metabolic changes were identified when only levels of the stimulating hormones were abnormal and the peripheral hormones were still in the reference range. Increased TSH and FSH levels appear to have additive effects on the development of NAFLD and to act independently from each other.

## 1. Introduction

Non-Alcoholic Fatty Liver Disease (NAFLD) is often considered the hepatic manifestation of Metabolic Syndrome (MetS) with Insulin Resistance (IR) as the main pathophysiological process [1]. NAFLD is defined as a fatty liver in patients with no alcoholic intake and where other diseases have been excluded [2]. The prevalence of NAFLD worldwide is 25% in the general population [3]. More recently, it was suggested to replace the term NAFLD with Metabolic dysfunction-Associated Fatty Liver Disease (MAFLD) [4]. The term MAFLD was suggested in order to include all stages of activity and fibrosis, and not to base a diagnosis on exclusion of another disease. MAFLD identifies patients with various stages of fatty liver disease and comorbidities, such as overweight/obesity, Type 2 Diabetes Mellitus (T2DM), and metabolic dysregulation [5]. However, there is an ongoing discussion about the advantage of using MAFLD over NAFLD, because MAFLD criteria exclude a specific population of NAFLD patients [6]. These MAFLD−/NAFLD+ patients were younger than MAFLD+/NAFLD+ patients. The vast majority of available studies are based on patients with a diagnosis of NAFLD.

NAFLD has been shown to be sexually dimorphic, and male individuals show more severe stages of NAFLD [7]. Hypothyroidism was identified as key element for the occurrence of NAFLD [8] but the association of both diseases was questioned by another study and suggests the contribution of additional factors, one of them being sex hormones [9]. This review compares the effects of Thyroid Tormones (THs) and sex hormones (estrogens, testosterone), including their respective stimulating hormones, Thyroid-Stimulating Hormone (TSH) and Follicle-Stimulating Hormone (FSH), on hepatic metabolism. Furthermore, the potential interaction of FSH and TSH in the dysregulation of hepatic metabolism will be discussed.

## 2. Non-Alcoholic Fatty Liver Disease

The development of NAFLD is strongly correlated with MetS and, depending on the criteria used for diagnosis, up to 90% of patients with NAFLD have MetS [10]. It is suspected that activation of Protein Kinase C (PKC)-ε by a high-fat diet, in combination with increased levels of fetuin B, selenoprotein P and Fibroblast Growth Factor 21 (FGF21) may lead to Insulin Resistance (IR) and hepatosteatosis [1]. On the other hand, IR contributes to liver damage. Several studies, however, suggest a disconnection between NAFLD and MetS because overexpression of Diacylglycerol O-Acyltransferase 2 (*DGAT2*) led to marked hepatosteatosis, whereas glucose and insulin levels remained normal. Similarly, inhibition of Very-Low-Density Lipoprotein (VLDL) secretion, choline deficiency, hyperbetalipoproteinemia and Lysosomal Acid Lipase (LAL) deficiency only caused hepatosteatosis but no changes in insulin sensitivity. NAFLD is defined as Fatty Acid (FA) infiltration of >5% hepatocytes and characterized by higher FA input (uptake from plasma or lipogenesis) than output (FA oxidation and secretion of triglycerides (TGs) as VLDL). It has been shown that TGs in the liver were derived to 59% from Free Fatty Acids (FFAs) in plasma, to 26% by de novo hepatic lipogenesis and to 15% by diet [10]. The disposition to develop NAFLD is thought to be due to priming. Both undernutrition during pregnancy and a high-fat diet have been linked to increased fat accumulation in the liver of offspring later in life. Accumulation of FFAs and TGs (steatosis) is regarded as the first hit in the pathogenesis of NAFLD. Lipophagy and VLDL production are impaired in NAFLD and increased β-oxidation of FAs leads to elevated intracellular Reactive Oxygen Species (ROS) in hepatocytes due to mitochondrial electron leakage [11]. The resulting hepatocyte damage together with inflammation, proliferation of hepatic stellate cells, fibrosis and necrosis leads to the progression of hepatosteatosis to Non-Alcoholic Steatohepatitis (NASH) [12]. Forty-three to forty-four percent of patients with NAFLD progress to NASH, which is prevalent worldwide in the general population at 1.5–6.5% [3]. Seven to thirty percent of NASH patients progress to liver fibrosis and the overall risk of hepatosteatosis progressing to fibrosis is 1–2% [12]. Further, there is the risk that patients with hepatic fibrosis develop hepatocellular carcinoma. Due to the potential progression of NAFLD to NASH, liver fibrosis and hepatocellular carcinoma, prevention of NAFLD is highly important. NAFLD is associated with alterations in various endocrine tissues, namely thyroid (hypothyroidism and decreased ratio of Thyroxine (T4)/Triiodthyronine (T3)), ovaries (increased or decreased estrogen and increased androgen levels) and testis (decreased testosterone levels), pituitary gland (increased adrenocorticotropic hormone and prolactin and decreased growth hormone levels), adrenal glands (increased glucocorticoid, androgen levels and activation of the renin–angiotensin–aldosterone system) and pancreas (T2DM) [13]. Altered levels of sex hormones (menopause of women) and THs are the most frequent changes in the endocrine system and their relevance for the development of NAFLD, alone or in combination with each other, will be discussed in the following sections.

## 3. Hepatic Metabolism and Insulin Resistance

**Figure 1:**
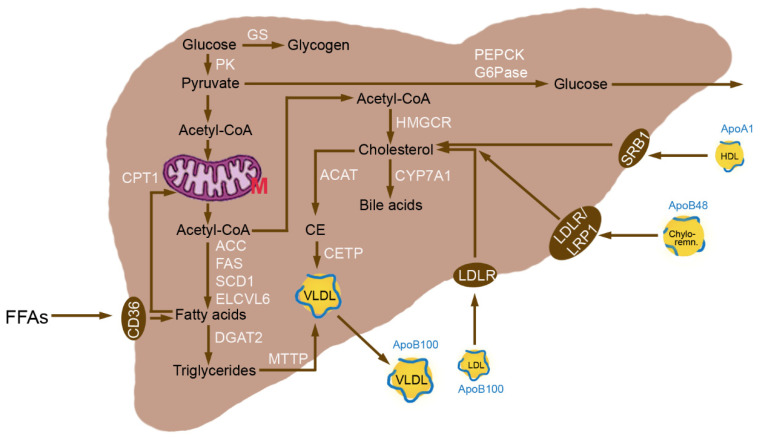
Enzymes relevant for the homeostasis of glucose, lipid and cholesterol homeostasis in healthy hepatocytes. Phosphoenolpyruvate Carboxykinase (PEPCK) converts phosphoenolpyruvate into oxaloacetate and is the rate-limiting step in gluconeogenesis. Pyruvate Kinase (PK) is the last step in glycolysis and transfers a phosphate group from phosphoenolpyruvate to pyruvate. Glucose may also be generated by Glucose-6-Phosphatase (G6Pase). The main role in glycogenesis is exerted by Glycogen Synthase (GS). Tricarboxylic Acid cycle (TCA) and β-oxidation of Fatty Acids (FAs) in the mitochondria serve for energy production. Activity of Carnitine Palmitoyl Transferase 1 (CPT1) determines the extent of β-oxidation. Lipogenesis is regulated through action of Acetyl-CoA Carboxylase 1 (ACC), Fatty Acid Synthase (FAS), Stearoyl-CoA Desaturase (SCD1) and Elongation of Very-Long chain fatty acids protein 6 (ELOVL6). Diacylglycerol O-Acyltransferase 2 (DGAT2) catalyzes the formation of Triglycerides (TGs) from diacylglycerol and fatty acid-CoA. Microsomal Triglyceride Transfer Protein (MTTP) is essential for the assembly with apoB100 in the synthesis of Very-Low-Density Lipoprotein (VLDL). Cholesterol may be synthesized de novo from Acetyl-CoA by β-Hydroxy-β-Methylglutaryl Coenzyme A Reductase (HMGCR) as rate-limiting step in the synthesis. Acyl-CoA Cholesterol Acyltransferase (ACAT) produces Cholesterol Esters (CE) that are integrated into VLDL by Cholesteryl Ester Transfer Protein (CETP). Cholesterol can also be metabolized to bile acids by the action of Cholesterol 7a-hydrolase (CYP7A1). Cholesterol may be taken up as Low-Density Lipoprotein (LDL) cholesterol by LDL Receptor (LDLR), as HDL cholesterol by scavenger receptor class B type 1 (SRB1) and as Chylomicron remnants (Chylo-remn.) by LDLR in humans and LDLR-related Protein 1 (LRP1) in rodents. Surface proteins are Apolipoprotein (Apo)B48 for chylomicron remnants, ApoB100 for VLDL and LDL, and ApoA1 for HDL cholesterol particles. Free Fatty Acids (FFAs) can be taken up by CD36, also termed Fatty Acid Translocase (FAT). Abbreviation: M, mitochondrion.

The liver plays a central role in glucose, lipid and cholesterol homeostasis and NAFLD is associated with dysregulation of several metabolic processes (Figure 1). To understand the development of NAFLD it is essential to consider the most important pathways in the regulation of lipid and cholesterol metabolism.

### 3.1. Normal Hepatic Metabolism

Glucolysis in the liver is controlled by the activities of Glucokinase (GK), Ghosphofructokinase (PFK), and Gyruvate Kinase (PK) [14]. In order to achieve gluconeogenesis, conversion of pyruvate to phosphoenolpyruvate is catalyzed by Pyruvate Carboxylase (PC) and Phosphoenolpyruvate Carboxykinase (PEPCK). The product from carboxylation of pyruvate, oxaloacetate, is an intermediate of the Tricarboxylic Acid cycle (TCA). Acetyl-CoA Carboxylase 1 (ACC) is the rate-limiting enzyme of FA synthesis and carboxylates acetyl-CoA to produce malonyl-CoA. Stearoyl-CoA Desaturase-1 (SCD1) is responsible for forming a double bond in stearoyl-CoA. Elongation of Very-Long Chain Fatty Acids Protein 6 (ELOVL6) is rate-limiting for the first FA elongation and transfers two carbons per cycle. DGAT2 catalyzes the formation of TGs from diacylglycerol and FA-CoA, which may be stored in the liver or be exported as VLDL via upregulation of Peroxisome Proliferator-Activated Receptor *(PPAR)-γ* and Microsomal Triglyceride Transfer Protein (*MTTP*) leading to increased plasma levels of TGs.

FA oxidation is conducted in mitochondria, endoplasmic reticulum and peroxisomes [15]. The first step is activation of FAs to fatty acyl-CoA by acyl-CoA synthetase in the outer mitochondrial membrane or endoplasmic reticulum. The long-chain FAs are shuttled as acyl-CoA into the matrix of the mitochondria by Carnitine Palmitoyltransferase-1 (CPT1), whereas medium- and short-chain FAs (up to C8) permeate the outer and inner mitochondrial membrane and are activated to acyl-CoA in the mitochondrial matrix. Autophagy and lipophagy mobilize TGs from lipid droplets [16]. Large lipid droplets are preferential substrates for Adipose Triglyceride Lipase (ATGL) and small lipid droplets for lipophagy. The released free FAs are transported into the mitochondria to undergo β-oxidation. The shortening of very-long-chain FAs (>16 carbon atoms) for further metabolization in mitochondria takes place in peroxisomes.

The synergistic actions of the transcription factors Sterol Regulatory Element-Binding Protein-1c (*SREBP-1c*) and Carbohydrate-Responsive Element-Binding Protein (*ChREBP*) coordinately activate the enzymatic machinery necessary for the conversion of excess glucose to FAs. SREBP-1c is the most relevant member of the family of the three transcription factors, namely SREBP-1a, SREBP-1c, and SREBP-2, in the liver [17]. All SREBPs act by translocation to the nucleus and binding to the target gene promoters. *ACC*, *FAS*, *SCD1*, Glycerol-3-Phosphate Acyltransferase (*GPAT*), and *Spot14* are controlled by *SREBP-1c* for lipogenesis. The main regulated proteins by SREBP-2 for cholesterol biosynthesis are β-Hydroxy-β-Methylglutaryl-CoA Synthase (*HMGCS*), β-Hydroxy-β-Methylglutaryl-CoA Reductase (*HMGCR*), Farnesyl Pyrophosphate Synthetase (*FPPS*), squalene synthase and Low-Density Lipoprotein (LDL) Receptor (*LDLR*). Acetyl-CoA is the smallest building block for de novo synthesis of cholesterol, which is regulated by HMGCR. Cholesterol and TGs, however, may also be taken up from the plasma as lipoproteins. The relative amount of protein, phospholipids, cholesterol and cholesterol esters and TGs varies between the lipoproteins [18]. VLDL have 60% TGs, 20% cholesterol + cholesterol esters, 15% phospholipids and 5% protein compared with 8% TGs, 50% cholesterol + cholesterol esters, 22% phospholipids and 20% protein in LDL and 5% TGs, 25% cholesterol + cholesterol esters, 30% phospholipids and 40% protein in High-Density Lipoprotein (HDL). There are also chylomicron remnants, which are taken up by the liver, consisting mainly of TGs and a variable amount of cholesterol and cholesterol esters [19]. VLDL and LDL are coated with Apolipoprotein (Apo)100, chylomicron remnants with ApoB48 and HDL with ApoA1 (Figure 1).

LDL cholesterol is taken up by the LDLR, HDL cholesterol through the Scavenger Receptor Class B type 1 (SRB1) and chylomicrons remnants by the LDLR in humans and by the LDLR-Related Protein 1 (LRP1) in mice [20]. Recycling of the LDLR through degradation in lysosomes and re-integration into the plasma membrane is an important mechanism to increase cholesterol uptake. Cholesterol can be excreted by secretion of unmodified cholesterol via the ATP-binding cassette subfamily G member 5/8 (ABCG5/8), after its transformation in bile salts by action of Cholesterol 7a-hydrolase (CYP7A1) through ABCB11 transporter and through VLDL secretion. In the latter process, cholesterol is esterified via Acyl-CoA Cholesterol Acyltransferase (ACAT) and integrated by the action of Cholesteryl Ester Transfer Protein (CETP) into VLDL. VLDL and chylomicron remnants transport TGs, cholesterol and cholesterol ester, whereas LDL and HDL carry cholesterol and cholesterol esters [21]. FFAs can be taken up by Fatty Acid Translocase (FAT), also termed CD36.

### 3.2. Insulin Resistance

Most of the above mentioned processes are regulated by insulin. The liver switches from producing glucose via glycogenolysis and gluconeogenesis in the fasted condition to the uptake of glucose with synthesis of glycogen, FFAs and TGs under the influence of insulin released after a meal. In the fasted condition, glucose, FAs and TGs are metabolized to acetyl-CoA for energy production in the TCA, and hepatocytes provide glucose for other organs.

Insulin binds to a plasma-membrane-associated receptor that is composed of α and β subunits. The β subunit is more specific for insulin binding and highly expressed in the liver [22]. The activation requires phosphotyrosine-binding scaffold proteins, usually insulin receptor substrate 1 (IRS1), which is the best described class of insulin scaffold proteins. Akt-signaling is central for hepatic signaling and substrates are Glycogen Synthase Kinase (GSK), transcription factor Forkhead Box O1 (FOXO1) and mammalian Target Of Rapamycin (mTOR). FOXO1 phosphorylation activates gluconeogenesis [23]. Insulin-induced phosphorylation prevents translocation of FOXO1 to the nucleus and downregulates genes required for gluconeogenesis, mainly *PEPCK* and Glucose-6-Phosphatase (*G6Pase*), which leads to decrease of hepatic glucose output. Insulin influences glucose and lipid metabolism by increased transcription of *SREBP-1c* and proteolytic cleavage of the membrane-bound SREBP-1c precursor to the active nuclear form. This enhances transcription of the genes required for FA and TG synthesis, mainly *ACC* and *FAS*. A consequence of increased FA synthesis is increased production of malonyl-CoA in the mitochondria resulting in inhibition of β-oxidation [24].

**Figure 2:**
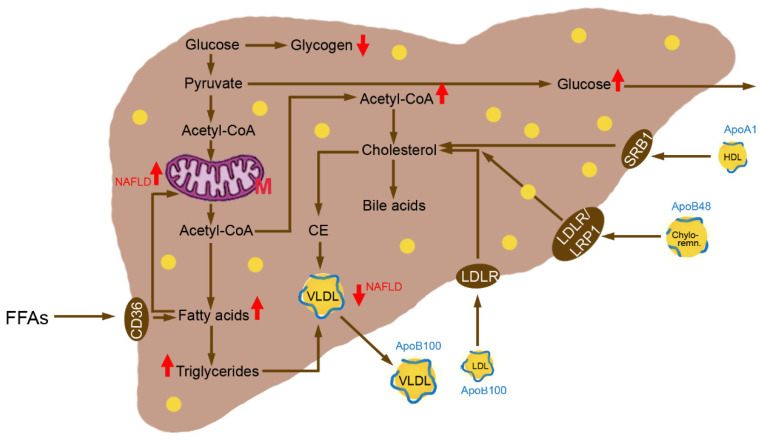
Changes in hepatic metabolism in insulin resistance and Non-Alcoholic Fatty Liver Disease (NAFLD). Glucose is metabolized to acetyl-CoA and used for synthesis of fatty acids and cholesterol. Free Fatty Acids (FFAs) are taken up by the CD36 receptor. The increased intracellular FA levels activate α-oxidation in the mitochondria. Due to the defective function of the respiratory chain in NAFLD, generation of reactive oxygen species is increased and induces inflammation. Excess Triglycerides (TGs) may accumulate in the hepatocytes or be exported into the blood. The export of TGs and cholesterol, however, is decreased in NAFLD due to impaired VLDL synthesis. Gluconeogenesis and hepatic output of glucose continues similar to the fasted condition. Abbreviations: Apo, apolipoprotein; Chylo-remn., Chylomicron Remnants; HDL, High-Density Lipoprotein; LDLR, LDL Receptor; LRP1, LDLR-Related Protein 1; M, Mitochondrion; SRB1, Scavenger Receptor Class B Type 1; VLDL, Very-Low-Density Lipoprotein.

Upon insulin resistance, the FOXO1 pathway is no more active and gluconeogenesis continues (Figure 2).

Excess lipid intermediates in TG synthesis, Diacylglycerol (DAG), phosphatidic acid and lysophosphatidic acid, cause insulin resistance by activating several serine/threonine kinases including PKC, mTOR, c-Jun N-terminal Kinase 1 (JNK-1) and Ikappa B (IkB) Kinase [25]. IkB kinase inhibits insulin signaling either directly through IRS-1 and IRS-2 serine phosphorylation or indirectly through mediation of transcription via Nuclear Factor Kappa B (NfkB). This results in reduced insulin activation of Phosphatidyl-Inositol-3-Kinase (PI3K) and Akt2. Reduced Akt activation leads to lower FOXO1 phosphorylation and increases transcription of *PEPCK* and *G6Pase*, which enhances gluconeogenesis and hepatic glucose output. The SREBP-1c pathway remains sensitive in insulin resistance and nuclear levels of the transcription factor are high. Hyperglycemia and hyperinsulinemia induce *SREBP-1* expression and activate Liver X Receptors (LXR) leading to transcriptional activation of lipogenic genes [25]. Hyperglycemia activates also *ChREBP*, which transcriptionally activates liver *PK* and all lipogenic genes. The combined action of SREBP-1 and ChREBP promotes lipogenesis and leads to malonyl-CoA production decreasing β-oxidation through inhibition of CPT1. Diacylglycerol (DAG)-mediated activation of PKC-ε impairs hepatic insulin signaling, thereby constraining insulin-stimulated hepatic glycogen synthesis [26]. Upon IR, FFAs entering the liver from the periphery, as well as those derived from de novo lipogenesis, will be preferentially esterified to TGs. The increased cellular amount of FFAs leads to upregulation of *CPT1* and downregulation of *PPAR-α* with increased oxidation of FAs [24]. By *PPAR-γ* and *SREBP-1c* downregulation combined with *SCD1* and *DGAT2* upregulation, TGs are synthesized.

Endocrine hormones such as THs and sex hormones act (partly) by modifying insulin sensitivity. It is supposed that hyperthyroidism causes mainly hepatic IR, whereas in hypothyroidism insulin resistance of peripheral tissue predominates [27]. Estrogens in women and androgens in men improve IR [28,29]. Data suggest that both TSH and FSH induce IR [30,31].

Glucose, TG and cholesterol metabolism are influenced by thyroid and sex hormones, including the respective stimulating hormones (Table 1).

## 4. Thyroid Hormones

### 4.1. Thyroid Diseases

The thyroid diseases hypothyroidism (overt and Subclinical Hypothyroidism (STH)) or hyperthyroidism (overt or subclinical hyperthyroidism), are the second most common endocrine diseases after diabetes mellitus. Hypothyroidism is estimated to affect 5% of the general population according to European prevalence estimates [32]. Meta-analysis of 17 studies reported prevalence data of 3.05% for hypothyroidism in Europe [33]. The majority of cases were STH, which is characterized by TSH of less than 10 mIU/L and normal TH levels [34]. Hyperthyroidism is less frequent than hypothyroidism and between 0.73–0.77% of the general population are affected in Europe [33]. Subclinical hyperthyroidism can be divided into two categories according to TSH levels: low, but detectable, TSH (0.1–0.4 IU/L) and less than 0.1 mIU/L [35]. In both categories, TH levels are in the normal range. Prevalence is most common in patients receiving levothyroxine (L-T4) replacement therapy and may be as high as 20%. Patients with multinodular goiter tend to have subclinical hyperthyroidism. Progression to overt hyperthyroidism is low (3% in women >60 y) in the group with detectable TSH values and higher (27%) in the group with TSH levels <0.1 mIU/L. In overt hypothyroidism TSH levels are markedly increased, whereas T3 and T4 values are decreased. Normal TSH serum levels are mostly given as 0.4–4.0 mIU/L.

Correlations of THs and TSH with metabolic dysregulation were found [36]. For instance, a linear association of TSH with total cholesterol, TGs, and LDL and HDL cholesterol was observed [37]. TG levels were positively associated with TSH and fasting glucose blood levels with T3 [36], whereas another study [38] reported positive correlation of TSH with TGs, free Thyroxine (fT4) with HDL-cholesterol, blood pressure with glucose and negative correlation of fT4 with total cholesterol. Association of reduced fT4 levels with visceral obesity and IR has also been reported in the literature and one study from Nigeria found correlation of fT4 and MetS [39]. The relationship of STH to metabolic dysregulation was strongest in the population of post-menopausal women, which suggests a synergistic effect of THs and sex hormones on the pathology. The differences between the studies may be due to factors, for instance age, sex, nutrition, ethnicity and physical activity.

### 4.2. Effects of THs on Hepatic Metabolism

THs increase oxygen consumption and catabolism of the energy sources in most organs (e.g., adipose tissue, skeletal muscle, liver) involved in glucose and lipid homeostasis. TH effects in the liver are mediated by activation of TH-Receptor β (TRβ) which is the main thyroid hormone receptor in the liver [40]. THs induce the expression of *G6Pase* and β2-adrenergic receptor and weaken the action of inhibitory G protein (Gi) [41]. This leads to stimulation of gluconeogenesis and glycolysis. Further, gluconeogenesis is increased by increase of PEPCK and PC activities. Decrease of Akt2 by THs prevents the stimulating effect of Glycogen Synthase (GS) in insulin-induced glycogen synthesis, but THs can also inhibit GS directly [42]. Akt (protein kinase B) proteins Akt1, Akt2 and Akt3 are serine/threonine proteins that are activated by PI3K [43]. Akt2 is the most closely related to glucose and lipid metabolism, is regulated by insulin and mainly distributed in liver, brown fat and skeletal muscle. Glucose Transporter (GLUT)2 is responsible for hepatic glucose output, and its stimulation by THs results in increased hepatic output of glucose. Lactate is transported from the peripheral tissues to the liver (Cori cycle), where it is converted by lactate dehydrogenase into pyruvate [44]. Gluconeogenesis from pyruvate represents an important source for glucose in hyperthyroidism. Further, amino acids from muscle (lactate, alanine, glutamine) serve as substrates for gluconeogenesis. Despite the effects of THs on enzyme expression, no differences in the glycolysis rate have been observed [42]. This suggests that the important effects of THs on energy metabolism occur at the level of the mitochondria (e.g., β-oxidation of FAs).

**Figure 3:**
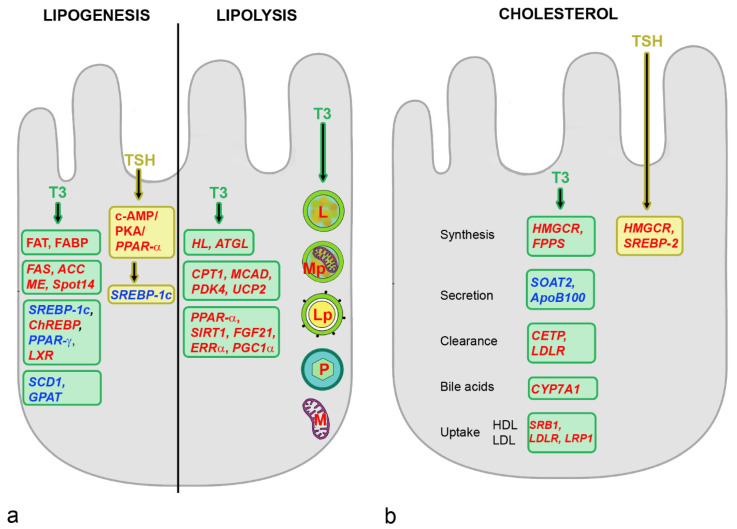
Influence of TH on lipid and cholesterol metabolism. (**a**): T3 stimulates lipogenesis by increase of Fatty Acid Translocase (FAT), Fatty Acid Binding Protein (FABP), upregulation of Fatty Acid Synthase (*FAS*), Acetyl-CoA Carboxylase *(ACC*), Malic Enzyme (*ME*), Spot 14 homolog (*Spot14*), decrease of Sterol Regulatory Element-Binding Protein 1c (*SREBP-1c*) and Peroxisome Proliferator Activated Receptor (PPAR)-γ combined with increase of Carbohydrate-Responsive Element-Binding Protein (*ChREBP*) and Liver X Receptor (*LXR*) and decrease of Stearoyl-CoA Desaturase (*SCD1*) and Glycerol-3-Phosphate Acyltransferase (*GPAT*). TSH acts on lipogenesis through upregulation of c-AMP, Protein Kinase A (PKA) and PPAR-α, which cause activation of *SREBP-1c*. T3 stimulates lipolysis by upregulation of Hepatic Lipase (*HL*) and Adipose Triglyceride Lipase (*ATGL*). β-Oxidation is activated by increased expression of *CPT1* and the mitochondrial enzymes, Medium-Chain Acyl-CoA Dehydrogenase (*MCAD*), Pyruvate Dehydrogenase Kinase isoform 4 (*PDK4*) and mitochondrial Uncoupling Protein 2 (*UCP2*). The *CPT1* expression can also be stimulated via PPAR-α, Sirtuin 1 (SIRT1), Fibroblast Growth Factor 21 (FGF21), Estrogen-Related Receptor α (ERRα) and PPARγ-Coactivator 1α (PGC-1α), to increase uptake of Fatty Acids (FAs) in the mitochondria. Further, synthesis of mitochondria, mitophagy, lipophagy, degradation of lipids in lysosomes and enzyme activity of peroxisomes are induced by T3. Abbreviations: L, lysosome; Lp, lipophagy; M, mitochondrion; Mp, mitophagy; P, peroxisome. (**b**): Cholesterol synthesis is affected by T3 at the level of synthesis by induction of β-Hydroxy-β-Methylglutaryl-CoA Reductase (*HMGCR*) and Farnesyl Pyrophosphate Synthase (*FPPS*), secretion by downregulation of Sterol O-Acyltransferase 2 (*SOAT2*) and Apolipoprotein 100 (*ApoB100*), clearance by upregulation of Cholesteryl Ester Transfer Protein (*CETP*) and Low-Density Lipoprotein Receptor (*LDLR*), excretion by upregulation of *CYP7A1* and uptake of HDL cholesterol by Scavenger Receptor Class B (*SRB1*) and of LDL cholesterol by *LDLR* and Low-Density Lipoprotein Receptor-related Protein (*LRP1*). Cholesterol levels are increased by TSH through upregulation of *HMGCR* and *SREBP-2*. Increased levels are indicated by red and decreases by blue color. Regulated genes are written in italics.

THs stimulate both liponeogenesis and lipolysis. THs act on TG synthesis via an increase of FAT and Fatty Acid Binding Protein (FABP) for influx and uptake of FAs [45] (Figure 3). Transcription of genes involved in lipogenesis, such as *FAS*, *ACC*, Malic Enzyme (*ME*) and SPOT 14 homolog (*Spot14*), is increased. THs decrease transcription factor *SREBP-1c* and increase LXR, both of which stimulate *ACC* expression [46]. All the genes encoding enzymes involved in lipogenesis, namely *FAS*, *ACC*, *SCD1*, *PK*, Glucose-6-Phosphate Dehydrogenase (*G6PD*), *ME* and *Spot14*, are direct ChREBP targets and THs increase expression by increasing ChREBP [47]. *SCD1* and Glycosyltransferase (*GTAT*) are downregulated to limit the amount of FAs stored as TGs in the liver. THs influence lipolysis by increasing hydrolysis via ATGL and Hepatic Lipase (HL) and lipid mobilization by increased expression of zinc α2 glycoprotein. THs can regulate both the number and expression levels of the peroxisomal enzymes but the mechanisms, by which thyroid hormones regulate peroxisome synthesis and function, are currently unknown [41]. Entry of FAs into mitochondria is stimulated by a TH-induced increase of *CPT1* transcription. The carrier can also be stimulated via the PPAR-γ Coactivator 1α (PGC-1α), Estrogen-Related Receptor-α (ERR-α), PPAR-α and FGF21. The contribution of FGF21 to hepatic metabolism includes increase of insulin sensitivity and glucose clearance and reduction of plasma TG levels, and T3 is able to increase its hepatic expression [48]. The enzymes of β-oxidation, Medium-Chain Acyl-CoA Dehydrogenase (MCAD), Pyruvate Dehydrogenase Kinase isoform 4 (PDK4) and mitochondrial Uncoupling Protein 2 (UCP2) are also stimulated by THs. Stimulation of synthesis of mitochondria by THs via PGC1α/Nuclear Respiratory Factor 1 (NRF1)/mitochondrial transcription factor A (mtTFA) signaling also serves to increase the metabolization of FAs. Mitochondrial enzymes may be regulated via a truncated form of the TH receptor in mitochondria by binding to mitochondrial DNA, but the mechanism is not really clear. The increased mitophagy serves to eliminate defective mitochondria and reduce cell injury by ROS generated by β-oxidation of FAs. Additional mechanisms decrease TG content and intensify lipolysis. Lipophagy is stimulated for increased mobilization of FAs from lipid droplets [41]. Stored lipid droplets are engulfed by autolysosomes and after autophagosome–lysosome fusion delivered to lysosomes for degradation. The process is most likely upregulated by THs because the number of lipid-laden autophagosomes in hepatocytes is increased by THs. Lysosomal biogenesis by inhibition of mTOR complex 1 and activation of Transcription Factor EB (TFEB) that coordinates expression of lysosomal hydrolases, membrane proteins and genes involved in autophagy contributes to the stimulation of lipophagy. Lysosomal activity is increased by Lysosomal Acid Lipase (LAL) expression. The mechanism to increase autophagy involves activation of NAD-dependent protein deacetylase Sirtuin 1 (SIR1), which decreases FOXO1 acetylation and phosphorylation. This leads to increased transcriptional activity and nuclear localization of FOXO. In summary, the action of THs on lipolysis is greater than on lipogenesis.

Regulation of cholesterol levels by THs is mediated by transcription of *HMGCR* and *FPPS* to increase cholesterol biosynthesis. Negative regulation of cholesterol secretion occurs by decrease of Sterol-O-Acyltransferase 2 (*SOAT2*) and *ApoB100*. Increased levels of CETP and LDLR lead to increased serum cholesterol clearance. LDLR-Related Protein (LRP1) to remove TGs from chylomicron remnants and VLDL are also increased in rodents. THs can decrease circulating Pro-protein Convertase Subtilisin/Kexin type 9 (PCSK9) levels, which contributes to lower LDL cholesterol levels by enhancing LDL receptor recycling. Cholesterol transport is stimulated by ApoA1 and SRB1 to increase cholesterol efflux from peripheral tissues by HDL. CYP7A1 activity for conversion of cholesterol to bile acids and ABCG5/8 to increase excretion of cholesterol are also stimulated.

The positive effects of TH on hepatic metabolism, especially the lowering of LDL cholesterol and of TG levels, suggested THs as a potential treatment for NAFLD. Thyromimetics binding to TRα and TRβ like 3,5-diiodothyronine, a metabolite of TH metabolism, and its synthetic analogue, TRC150044, were studied for this application. TRC150044 showed beneficial effects on lipid metabolism in the absence of adverse effects on other TH-responsive tissues in preclinical studies. In the phase I clinical trial NCT01408667, however, administration of TRC150044 did not change intrahepatic TG levels [49]. To avoid the unwanted side effects of THs on the heart and the hypothalamus–pituitary–thyroid axis, TRβ-selective ligands were developed. The first compounds, GC-1, GC-24, KB141, KB2115, MB07344 and MB07811, reduced LDL cholesterol and decreased hepatosteotosis in animal experiments. However, none of these compounds have so far developed into therapeutics. Sobetirome (GC-1) was stopped after phase I clinical trial due to lack of funding, whereas Eprotirome (KB2115) was stopped because chondrotoxicity was observed in long-term studies in dogs [50]. A phase II clinical trial (NCT 04173065) for evaluation of the efficacy of VK2809 (MB07811) is listed as recruiting since 2019. Resmetirom (MGL-3196) is a more recently developed selective TRβ agonist. Decreased LDL cholesterol, ApoB and TG levels were reported in a phase II clinical trial and a phase III clinical trial (NCT 03900429) is ongoing [51]. The ASC41 prodrug, which is activated to ASC41-A by the CYP3A4 isoenzyme, reduced blood levels of LDL cholesterol and TGs, hepatosteatosis and inflammation in a phase I clinical trial in China [52]. The most recently developed TRβ-selective ligands are IS25 and its prodrug TG68, which displayed mitotic effects in hepatocytes and increased the regenerative potential of the liver in animal experiments [53].

### 4.3. Effects of TSH on Hepatic Metabolism

TSH levels increase with age and are higher in females than in males. A study from the US reported that the median of TSH in the healthy population increased from 1.28 mU/L at age 20–29 years to 1.99 mU/L at age 80 years and older [54]. The lower limit decreased, and the upper limit increased with age, and the median shifted to higher values. Women had higher median TSH values than men, but the difference was small and varied with age. The age-related increase in TSH was interpreted as a changed set point or reduced bioreactivity rather than thyroid disease and seen as the cause for the decrease of free Triiodothyronine (fT3) levels after 60 years in women [55]. Until that age, fT4 levels were the same in males and females and the fT3/fT4 ratio remained stable. Only at higher ages, there was a rapid decline in fT3 levels in women.

Hepatocytes express the TSH receptor and TSH promotes lipogenesis, gluconeogenesis and decreases bile acid synthesis. TSH increases gluconeogenesis through increased expression *PEPCK* and *G6Pase* [56]. The effect is mediated by upregulation of the cAMP/Protein Kinase A (PKA)/cAMP-Responsive Element Binding Protein (CREB) pathway [57]. According to studies in rodents, TSH induces hepatosteatosis by increase of lipogenesis via SREBP-1c [41] (Figure 3). The decreased ATP-Activated Protein Kinase (AMPK) activity increased the expression of genes associated with lipogenesis and cholesterol biosynthesis. Other effects of AMPK, such as stimulation of glucose uptake and glycolysis and reduction of gluconeogenesis and lipogenesis, were also decreased [58]. The AMPK-induced increase in mitophagy, autophagy and oxidative metabolism was abolished. Decreased lipolysis and β-oxidation are mediated by downregulation of the PPAR-α pathway [57,59].

LDL cholesterol uptake is decreased by increase of PCSK9. PCSK9 binds to the LDLR on the cell surface and after internalization of the receptor, it inhibits endocytic recycling of the LDLR, resulting in the degradation of both LDLR and PCSK9 in lysosomes. The reduction of LDLR decreases the uptake and results in higher LDL cholesterol plasma levels. Intracellular accumulation of cholesterol results from a decrease in CYP7A1 through the SREBP-2/Hepatocyte Nuclear Factor 4α (HNF4α)/CYP7A1 signaling pathway, which leads to decreased bile acid synthesis [41]. Serum bile acid concentrations were inversely correlated with TSH levels and this association was stronger in individuals <65 years than in older subjects. It should be mentioned that decreased THs and increased TSH levels act controversial on cholesterol levels, low TH levels decrease cholesterol synthesis, whereas increased TSH levels increase it [60]. TSH levels in the euthyroid range were associated with total cholesterol, LDL cholesterol and TGs and inversely with HDL cholesterol. The relationship was stronger for overweight individuals.

### 4.4. THs and NAFLD

The risk for NAFLD is increased in hypothyroid patients in a dose-dependent manner. A linear correlation of TSH levels and risk for NASH and a correlation of high fT3 or low fT4 plasma levels (both within the reference range) to hepatosteatosis were found [61]. Possible mechanisms include direct effects on LPL activity leading to increased TG and cholesterol levels [48]. However, the link between hypothyroidism and NAFLD has not been reported in all studies and the underlying mechanism is not entirely clear. Independent risk factors for NAFLD in hypothyroidism and euthyroid patients are fT3/fT4 ratio, IR, waist circumferences and level of TGs. The balance between hepatic lipid synthesis and catabolism may be disrupted by abundance of dietary lipids and glucose, which enhances glycolysis, lipogenesis and levels of free FAs. In hypothyroid rodents, glycogen accumulation was increased and lipogenesis decreased due to reduced activity of FAS and ACC [62]. These effects could be reversed by administration of T3 to the hypothyroid animals and, based on these experiments, the authors concluded that hypothyroidism per se may not induce hepatosteatosis. Hypothyroidism, however, may increase the risk of liver fibrosis because low fT3 values correlated with liver stiffness and increased fibrosis score. Studies indicated that, after adjustment for insulin resistance, sex and steatosis parameters, low fT3 levels were an independent risk factor for advanced fibrosis [63].

TSH has been reported to be an independent risk factor for NASH [64,65]. Further, in a meta-analysis TSH levels were positively correlated with NAFLD, independent of TH levels, and hypothyroid patients are 2.1 times more likely to develop NAFLD and 3.6 times more likely to develop NASH [47]. Both diseases were more prevalent in patients with overt hypothyroidism and STH. Not only TSH levels outside the normal range but also increased TSH levels within the normal range have been associated with NAFLD. The effect of hypothyroidism appears to depend on additional factors, mainly the menopausal status in women. Hypothyroidism induced peripheral obesity in pre-menopausal women and visceral fat deposition in post-menopausal women [66]. Hypercholesterinemia was more pronounced in peri-menopausal than in pre-menopausal women, potential reasons are that intrahepatic deiodinase expression is defective in NASH and hepatic FAs in NAFLD impair TH receptor activity [59]. The correlation of raised TSH levels with progression of NAFLD was not seen in all studies. The age-dependency in females further suggests a potential contribution of sex hormones and FSH.

Compared with hypothyroidism, the effects of hyperthyroidism on NAFLD have not been studied systematically. According to one case study of Graves’ disease and NASH, however, high TH levels were beneficial for the liver condition [67].

## 5. Sex Hormones

### 5.1. Effects of Estrogens on Hepatic Metabolism

Data from animal models, namely the obese spontaneously hypertensive rat and the C57Bl6/J HFD mouse, suggest prominent sexual dimorphism of several hepatic genes [68]. Expression of *JNK* and *AMPK*, insulin sensitivity and FA oxidation are higher in women than in men. Further, FA uptake is increased by higher CD36 levels in female compared with male livers. The accumulation of lipid droplets, on the other hand, is higher in male livers due to PPAR-γ-induced activation of *SREBP-1c*. The stronger downregulation of aquaporin 9 (*AQ9*) leads to decreased glycerol uptake in female livers. In combination, these effects lead to a lower propensity for hepatosteatosis in females.

Changes in sex hormones (estrogens, progesterone and testosterone) and gonadotropins (FSH, Luteinizing hormone/Interstitial Cell Stimulating Hormone (LH/ICSH)) are associated with normal aging, but levels change more in women than in men. Around the world, a certain variation in the average age for menopause was observed (https://www.statista.com/statistics/1242217/womens-age-perimenopause-onset-worldwide-by-country/, accessed on 10 June 2022) but until the age of 50 years around 80% of women had experienced peri-menopause symptoms. In Asian countries, >50% of women reported irregular menses of post-menopause at the age of 49 years [69]. After menopause, estradiol (E2) and FSH levels change markedly from 30–400 pg/mL E2 and 4–12 mIU/mL FSH in menstruating women to <30 pg/mL E2 in menopausal women and an increase of FSH to >40 mIU/mL (https://www.breastcancer.org/treatment-side-effects/menopause/types/testing, accessed on 29 May 2022).

Estrogens act on Estrogen Receptors (ERs), including classic nuclear receptors ERα and ERβ, and membrane-bound receptors, G Protein-coupled ER (GPER, also known as GPR30) and membrane-associated ERα and ERβ variants [70]. ERα is the primary ER in insulin-sensitive tissues for genomic regulation by estrogens but E2 signals can also be non-genomically transmitted via GPER. Only membrane-localized ERα was necessary to decrease cholesterol, TG and FA content in mouse models. By contrast, ERβ has lipogenic and diabetogenic function [71]. Levels of Sex Hormone-Binding Globulin (SHBG), mainly produced in the liver, determine the availability of estrogens and androgens [28]. An interaction with thyroid metabolism exists in the way that THs regulate SHBG production by HNF-4α in response to changes in the metabolic state of the liver [72]. There was no sex-specific association but men with NAFLD had lower testosterone and estradiol levels than men without NAFLD [73]. The mechanisms for this association are currently not identified.

Estrogens reduce gluconeogenesis and increase glycogen synthesis and storage in the liver, lowering circulating glucose levels [71]. Glucose metabolism is influenced through estrogen-induced expression of glucose transporters, mainly GLUT2 [74]. Further, activity of most enzymes involved in glycolysis can be upregulated. Estrogens also increase activity of the first enzymes in the TCA, namely citrate synthase, aconitase and isocitrate dehydrogenase. The effect could be due to the increased insulin sensitivity of the liver, which may explain why ERα-KO mice have decreased insulin sensitivity up to IR [28]. In experiments with ovariectomized rodents, increased glucose levels were seen that correlated with increased glucagon signaling due to increased glucagon receptor expression [71]. E2 could not reverse the glucose imbalance, potentially because progesterone was missing. In addition to the positive effect of E2 on glucose metabolism, mainly by ERα signaling, estrogens could also act by stimulation of PFK and glycolysis, as has been observed in MCF-7 ER+ breast carcinoma cells [75]. Estrogens limit the release of serum FAs from the adipose tissue in response to insulin and promoted FA oxidation in skeletal muscle [76]. The limited FA delivery to the liver may contribute to the prevention of NAFLD.

**Figure 4:**
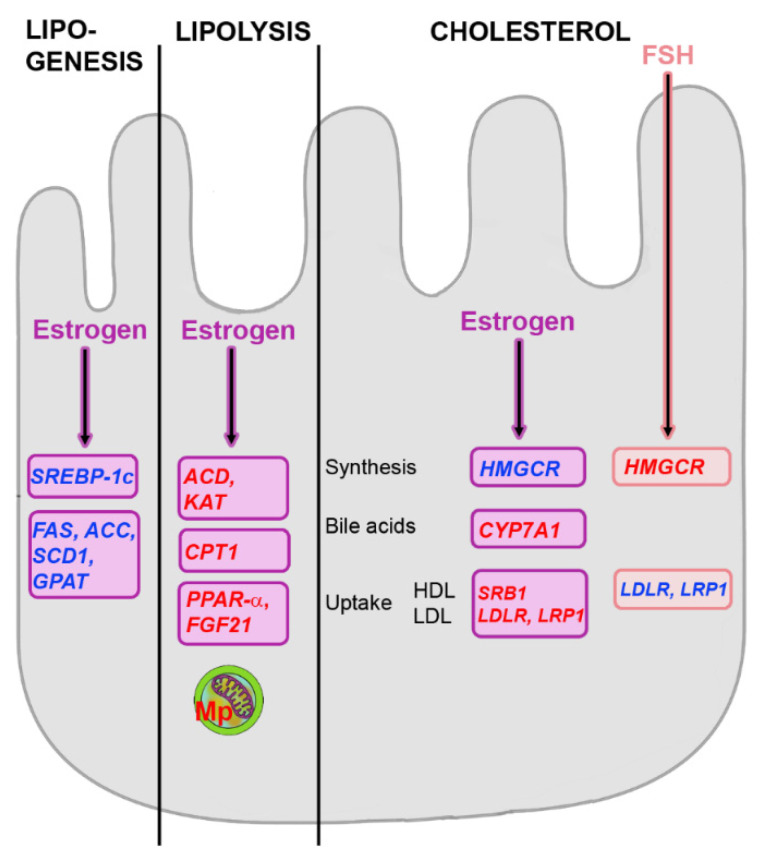
Changes in lipogenesis, lipolysis and cholesterol under the influence of estrogens and Follicle-Stimulating Hormone (FSH). Levels of Sterol Regulatory Element-binding Protein (*SREBP-1c*) and enzymes of Fatty Acid (FA) synthesis, Fatty Acid Synthase (*FAS*), Acetyl-CoA Carboxylase (*ACC*), Stearoyl-CoA Desaturase (*SCD1*) and Glycerol-3-Phosphate Acyltransferase (*GPAT*) are decreased. Estrogens increase lipolysis by stimulation of Acyl-CoA Dehydrogenase (*ACD*), 3-Ketoacyl-CoA Thiolase (*KAT*) and Carnitine Palmitoyl Transferase 1 (*CPT1*). Further, Peroxisome Proliferator-Activated Receptor (*PPAR*)-α and Fibroblast Growth Factor (*FGF*)21 levels and Mitophagy (Mp) are increased. Estrogens decrease cholesterol synthesis by downregulation of β-Hydroxy-β-Methylglutaryl Coenzyme A Reductase (*HMGCR*). Upregulation of Cholesterol 7a-hydrolase (*CYP7A1)* increases transformation of cholesterol into bile acids. Uptake of cholesterol is increased by upregulation of Scavenger Receptor Class B (*SRB1*) and Low-Density Lipoprotein Receptor (*LDLR*) or Low-Density Lipoprotein Receptor-related Protein (*LRP1*). FSH increases cholesterol synthesis by upregulation of HMGCR and decreases uptake by downregulation of *LDLR* or *LRP1*. Increased levels are indicated by red and decreases by blue color. Regulated genes are written in italic.

FFA uptake and de novo lipogenesis are decreased by estrogens, whereas VLDL secretion and β-oxidation in the mitochondria is stimulated [77] (Figure 4). Estrogen treatment suppressed *FAS*, *SCD1* and the mitochondrial form of *GPAT1*, the first step in glycerolipid synthesis, to decrease lipogenesis [78]. To increase lipolysis, activities of Acyl-CoA Dehydrogenase (ACD) and 3-Ketoacyl-CoA Thiolase (KAT), the first and last step of β-oxidation, respectively, are upregulated [74]. Upregulation of CPT1 and improvement of mitochondrial function, e.g., by mitophagy, are further mechanisms to increase lipolysis. Estrogens also stimulate FA oxidation indirectly by enhancing FGF21 production [79].

Estrogens decreased LDL cholesterol and increased HDL cholesterol levels in blood, although increased HDL receptor expression was detected in peripheral tissues [80]. In the liver, increased reverse cholesterol uptake was reported. This may be due to similar effects as in peripheral tissue, namely the upregulation of SRB1 for HDL cholesterol, and by increased expression of LDLR for LDL cholesterol [78]. According to animal studies, cholesterol synthesis is increased by estrogens in peripheral tissues through stimulation of HMGCR activity, whereas in the liver HMGCR is reduced due to decreased *SREBP-2* expression [76]. Conversion of cholesterol into bile acids is increased by estrogens and can result in gallstone formation [81]. Animals with estrogen deficiency do not increase cholesterol synthesis but decrease cholesterol conversion into bile acids by reducing CYP7A1 activity [70]. Export from the liver as HDL cholesterol is also decreased.

Interpretation of estrogen effects is complicated by the fact that there are several open questions regarding the metabolic effects of estrogens. It is for instance not clear why estrogens produced by the ovary and white adipose tissue differently affected IR, as reported in a study including obese and lean post-menopausal women. The obese women with estrogen production in the adipose tissue had higher E2 values and more pronounced IR than lean post-menopausal women [28]. Another unclear point is that hormone replacement therapy by oral administration increases VLDL production by 80% leading to TG-rich dyslipidemia, whereas transdermal estrogen treatment did not show this effect [76].

### 5.2. Effects of Testosterone on Hepatic Metabolism

There is only a gradual decline in testosterone values of ~2%/year and an increase of ~3%/year in FSH values in aged men [82]. A systematic literature review including 97 studies found that 2.1 to 12.8% of middle-aged to older men had testosterone levels <300 ng/dL, corresponding to hypogonadism [83]. These data suggest that pronounced age-dependent hormonal changes in men compared with aging women affect only a small population.

Most data on the effects of androgens stem from rodent studies, where more prominent hormonal changes were induced. Testosterone-deficient rats had increased glucose synthesis and symptoms similar to T2DM and MetS [71]. Testosterone acts on glucose metabolism by increase of GLUT4 in peripheral tissues, insulin receptors and Insulin Receptor Substrate 1 (IRS-1) expression, resulting in increased insulin responsiveness [84]. In the liver, testosterone increased GLUT2 mRNA expression. Androgen replacement therapy had positive effects by increasing expression of enzymes involved in glycolysis such as PFK and hexokinase [59].

In contrast to estrogens, androgens do not influence hepatic FA uptake [77]. In male rats, androgen deficiency caused higher serum and liver levels of TGs, cholesterol and LDL and increased levels of SREBP-1 and 2 [59]. Testosterone decreased lipogenesis by downregulating *ACC* and *FAS* and reducing *SCD1* expression, and decreasing hydrolysis of TGs by increasing lipoprotein lipase activity [71,84]. The combination of both effects led to decreased FA levels in hepatocytes. Androgen effects in obese mice included increase of *PPAR-α* expression, leading to increased FA oxidation and decreased lipogenesis through lowered SREBP-1c [85].

Effects of androgens on cholesterol metabolism were reported controversially. Lack of testosterone increased PCSK9 levels, resulting in decreased LDLR recycling and in increased LDL cholesterol blood levels [86]. Data from testosterone administration to castrated animals further showed that testosterone upregulated ApoE to increase cholesterol uptake and downregulated *CYP7A1* for bile acid synthesis [87]. In a clinical study evaluating testosterone effects in healthy volunteers, the hormone increased cholesterol synthesis by increasing *HMGCR* activity [88]. Although intracellular cholesterol levels were increased in both studies it is unclear, which mechanism could be more relevant for the physiological effects in humans because both models, administration to men with normal testosterone levels and to animals without any testosterone production, do not reflect the situation in elderly men.

### 5.3. Influence of FSH on Hepatic Metabolism

FSH values in women increase from about 4.7–21.5 IU/L in the reproductive phase to 25.8–134.8 IU/L after menopause. FSH levels rise abruptly in the early menopausal transition and are accompanied by alterations in bone remodeling, body composition and energy metabolism, all of which are most prominent during the late peri-menopause [89]. FSH levels of healthy adult men are 1.5–12.4 IU/L (https://www.medicalnewstoday.com/articles/317746, accessed on 11 May 2022). FSH levels increase age-dependently also in men but are in the low normal range in the low testosterone condition (secondary hypogonadism) [90]. Levels of the gonadotropin LH in post-menopausal women are in the same range as that of pre-menopausal women, but the rhythmicity of the levels is lost.

The FSH receptor is a G protein-coupled transmembrane receptor expressed on hepatocytes. To study the effects of FSH alone, ovariectomized mice supplemented with E2 and treated with FSH were used [91]. The authors reported that FSH increased fasting glucose by transcription of *Pepck* and *G6pase*. Gluconeogenesis was increased through increased translocation of G protein-coupled Receptor Kinase 2 (GRK2) to the membrane and enhanced nuclear translocation of cAMP-regulated transcriptional coactivator 2. It may be hypothesized that levels or sensitivity of FSH receptors play a role in metabolic dysregulation because raised FSH levels in pre-pubertal women predicted sensitivity for MetS in later life [92]. Studies identified a role of FSH in fasting hyperglycemia by using FSH receptor knockout mice [91] and the link between FSH levels and insulin resistance has been confirmed in post-menopausal women [31]. The relationship between IR and FSH levels may be mediated through regulation of Glucocorticoid Receptor (GCR) expression. This hypothesis is based on the fact that FSH upregulated GCR expression in the hepatocytes of hypogonadal mice [93].

FSH levels in post-menopausal women were not associated with dyslipidemia and only weakly with TG levels [94]. By contrast, association of FSH with total cholesterol and LDL cholesterol, especially in young post-menopausal women, was strong. Even slightly increased FSH levels were associated with higher serum cholesterol concentrations [95]. In post-menopausal women, higher FSH levels were associated with higher total cholesterol and LDL cholesterol in one study, whereas FSH levels were inversely associated with LDL cholesterol in another study [96]. Women with Premature Ovary Insufficiency (POI), who also present elevated FSH levels, had higher total cholesterol, HDL cholesterol and LDL cholesterol levels compared with healthy women [95]. Data from women with POI, compared with age- and Body Mass Index (BMI)-matched controls, showed higher TG and significantly lower HDL cholesterol levels. In another study, higher total cholesterol and LDL cholesterol were detected in POI women but no difference in HDL cholesterol and TG levels were seen. Finally, a meta-analysis of 458 patients with POI and 551 control persons showed a significant increase of total cholesterol, LDL cholesterol and TGs in POI patients compared with healthy controls and no differences in HDL cholesterol [97]. In that study, FSH stimulated lipogenesis and lipid droplet formation only in adipose tissue, not in the liver. Although one study did not report differences in fasting glucose, total cholesterol, HDL and LDL cholesterol or TG levels in women with raised FSH levels, most studies concord that cholesterol levels are increased when FSH levels are raised [96].

The close link between FSH and cholesterol levels makes inhibition of FSH signaling a potentially interesting target for hypercholesterolemia in menopause [98]. Significant improvement in TG and cholesterol levels in women under hormone replacement therapy was noted when FSH levels were reduced by more than 30% [99]. Signaling via FSH receptors is hypothesized to downregulate LDLR, which has a similar effect to decreased LDLR recycling. FSH was shown to reduce LDL resorption and induce cholesterol production [98]. FSH binding inhibited LDLR expression and at a threshold of 78.3 IU/L FSH significantly higher serum LDL and total cholesterol levels were measured than at lower FSH levels [99]. To exclude overlap of FSH and estrogen effects, data from pre-menopausal and peri-menopausal women with similar estrogen levels but significantly higher FSH levels in the peri-menopausal groups were analyzed [98]. The authors found that elevated FSH alone could induce cholesterol biosynthesis via upregulating HMGCR expression and activity.

### 5.4. Sex Hormones and NAFLD

Overall, the prevalence of NAFLD is higher in adult men than in women. In aged women, however, there is a marked increase in NAFLD prevalence from 42.9% in pre-menopausal obese women to 60.2% in post-menopausal women [77]. Estrogens are thought to protect against NAFLD by decreasing gluconeogenesis and lipogenesis and increasing cholesterol excretion and their reduction in menopause may partly explain the increase in prevalence from 32% to 60% mentioned in that study [59]. The association is confirmed by the reported reduced risk of NAFLD and fibrosis in women with hormone replacement therapy. Further, the increased prevalence of NAFLD upon long-term therapy with tamoxifen in breast cancer patients supports this theory [77].

The fact that changes in lipid and cholesterol levels were altered when estrogen levels were still in the normal range suggests that the rise in FSH is more important than the estrogen levels. This was the result of the Study of Women’s Health Across the Nation (SWAN) performed in the United States [100]. This study, including a large cohort of peri-menopausal women (42–52 years of age), found that menopausal transition occurred at the age of 47 years. The authors examined body fat in relation to endogenous hormone levels at various stages of the peri-menopause and post-menopause. High FSH levels were associated with increases in waist circumference, waist–hip ratio and visceral fat volume [100]. Other sex hormones may also play a role because NAFLD prevalence was not increased in women without hyperandrogenemia [59]. Lipid profiles in Polycystic Ovary Syndrome (PCOS) patients, who usually have increased androgen levels, were abnormal with low HDL cholesterol, high LDL cholesterol and high TG levels. In this pathology, estrogens are in the normal range, whereas total and free testosterone levels are usually increased. It may be possible that normal E2 levels are the result of conversion of testosterone to E2. Depending on the criteria that were used for the diagnosis of PCOS, the prevalence of NAFLD was indicated as 14.5–54.5% or 23.8–36.8% in these patients [101]. The prevalence of NAFLD was reported to range between 36–70% in another study including pre-menopausal women with PCOS [102], which was significantly higher than the prevalence of 14–34% in healthy women reported in this study. There was, however, no increase in NAFLD in healthy women taking oral contraceptive pills, excluding that the altered (usually decreased estrogen) hormone levels in users of oral contraceptive play a role in NAFLD development. Effects of progesterone on liver metabolism are poorly studied but human data suggest a proinflammatory effect of progesterone on NAFLD [103].

In contrast to women, androgens have positive effects on hepatosteatosis in men. Low testosterone levels were identified as a risk factor for NAFLD in men, whereas E2 acted protectively [104]. In another study, estrogens had a protective role against NAFLD in healthy men (20–60 y), whereas reduced testosterone levels played no role [105]. A US American study reported low total testosterone and high free E2 as a risk factor for NAFLD in men, whereas total E2, SHBG and free testosterone were not associated with NAFLD [106]. Data were adjusted for alcohol, smoking, age, race, physical activity, waist circumference and steroid hormones. It was hypothesized that there is a concentration range for both sexes, corresponding to decreased levels of androgens in men and increased levels in women, where metabolic dysfunction results. This concentration range was termed the “valley of death” [102]. Another explanation may be the different expression of the receptors in men and women. Low testosterone levels in men increased visceral adiposity, worsened insulin resistance and were associated with NAFLD [77].

The role of FSH in NAFLD in men was studied in Chinese men (80–98 y), out of which 24.3% had NAFLD. FSH was negatively correlated with total testosterone and E2 and positively with LH and liver enzymes [104]. The association of FSH levels with NAFLD in the aged men was independent of age, hypertension, diabetes mellitus, BMI and other sex hormones. FSH promoted fat accumulation in aging and fat redistribution from subcutaneous to visceral fat but appeared to play no role in the progression of NAFLD because there was no correlation to fibrosis. A correlation of FSH with obesity was observed in both sexes, and a protective role of low FSH has been seen in women. There was, on the other hand, one study that identified low FSH levels as a risk factor for NAFLD in women [107]. In addition to the absolute levels, the diurnal rhythm of FSH level also plays a role in NAFLD development. It was found that a normal circadian rhythm of FSH blood levels was associated with a higher incidence of NAFLD in post-menopausal women [108]. Secretions of stimulating hormones are timed in a hormone-specific circadian pattern, where FSH is higher in the afternoon [109]. By contrast, TSH levels are highest between midnight and early morning [110]. Rodent studies reported that FA and TG synthesis, lipolysis and glycogen storage occur during the active period, whereas glycogenolysis and hepatic gluconeogenesis are highest at the end of the sleeping phase [111]. It could be hypothesized that increased FSH levels primarily affect lipid metabolism, whereas elevated TSH levels affect mainly glucose metabolism.

## 6. Combined Action of TSH and FSH

### 6.1. Interaction on Hepatic Metabolic Homeostasis

Interactions between the thyroid and the reproductive system may occur at different levels. It has been observed in the hormone replacement therapy of post-menopausal women that oral estrogen therapy raises the circulating levels of Thyroxine-Binding Globulin (TBG) by altering its clearance, thereby increasing the bound T4 fraction and decreasing fT4 [112]. However, in women with normal thyroid function, the free T4 concentration can be replenished by elevated T4 concentrations that compensate for the decrease in the free fraction. Interaction of THs and sex hormones on a cellular basis may occur in all cells possessing both receptors, but mainly the effects on cells of the reproductive system have been studied [113]. Women suffering from hypothyroidism had decreased estrogen levels and increased circulating Gonadotropin-Releasing Hormone (GnRH).

A prevalence of STH in 14% of PCOS women compared with only 1% in the healthy controls included in this study was reported [30]. In this study, TSH levels correlated with IR and cortisol levels in obese PCOS women, and TSH was inversely correlated with E2 and SHBG in these women. In non-obese women, TSH correlated only with waist to hip ratio values. On the same line, Hashimoto’s thyroiditis was more common in PCOS patients than in the general population (27% versus 8%) [114]. These women had higher insulin levels and greater IR and higher total cholesterol and TSH levels, whereas fT4 and the fT4/fT3 ratio were significantly lower. IR was present when TSH levels of >2 mIU/L were measured. Patients with PCOS and Hashimoto’s thyroiditis had a higher BMI, fasting glucose, total cholesterol and IR than women with Hashimoto’s thyroiditis alone. Compared with euthyroid PCOS patients, women with STH and PCOS had higher TG, fasting insulin and IR but similar total cholesterol, HDL cholesterol and LDL cholesterol levels. These observations confirmed results of an earlier study that reported increased TSH levels in 21.4% of PCOS patients [115]. In this subgroup, increased fasting insulin, IR and total cholesterol/HDL ratio and lower HDL, fT4 and insulin sensitivity were determined.

### 6.2. Relation between TSH and FSH

Associations of TSH and FSH in post-menopausal women have been reported inconsistently. In one study, such a correlation was observed, whereas it was not reported in another [116,117]. STH was correlated with obesity and hypertriglyceridemia in pre-menopausal women [66]. In post-menopausal women this correlation was lost, suggesting that the influence of the high FSH levels was stronger than that of the high TSH levels. A cross-sectional study including 53,230 women aged 40 years or older studied the influence of raised FSH levels on hypothyroidism [118]. The authors found that the prevalence of hypothyroidism in women was increased in peri-menopause to a similar extent as in post-menopause after adjustment of age, age of menarche, year of examination, parity, educational level, smoking, alcohol, physical activity and BMI. Dyslipidemia was observed in 18% of peri-menopausal women, who on average had TSH levels of 7.56 ± 3.54 mIU/L [119]. Total cholesterol was positively linked to TSH levels and association with menstrual abnormalities was seen in 68% of patients with STH compared with 12% of the euthyroid controls. An Indian study reported a similar incidence of 68.2% in hypothyroid and 12.2% in euthyroid women together with a positive correlation of TSH with BMI [120].

To explain the higher prevalence of STH in post-menopausal women, cross-reactivity between TSH and FSH might be postulated [121]. Although FSH and TSH receptors share 40% amino acid sequence identity, only a weak interaction has been found. It was, on the other hand, reported that anti-TSH antibodies could stimulate FSH because such patients needed less stimulation with FSH receptor agonists in in vitro fertilization treatment [122]. Hypothyroidism increases Thyrotropin Releasing Hormone (TRH) transcription and secretion from the hypothalamus [123] but in healthy pre-menopausal women hypophyseal stimulation by TRH of only TSH, LH and prolactin, but not of FSH, was observed [124]. GnRH levels did not show prominent changes in peri-menopausal and post-menopausal women compared with pre-menopausal women, and only GnRH pulse frequency was decreased in the post-menopausal women [125]. It is, therefore, not expected that GnRH levels play a role in the association of menopause and STH.

TSH and FSH could also be linked through obesity. Despite the fact that a link between high TSH and BMI has been reported, the percentage of overweight or obesity in patients with high TSH levels in hypothyroidism (76.5%) and with low TSH values in hyperthyroidism (58.8%) were not dramatically different [126]. The link of FSH and body weight was inverse and 20% of obese women had FSH <30 IU/L one year after menopause compared with 2% in women with normal weight [127]. This makes obesity as an important common link between TSH and FSH actions unlikely. However, both hormones may act through leptin because the association of TSH levels and leptin was independent from BMI and FSH also increased leptin levels [128,129].

Consistent with the lack of a common mechanism, TH and estrogen replacement therapy improved metabolic dysregulation and lowered the prevalence of NAFLD. A decrease of NAFLD from 39.9% in untreated women to 24.4% upon treatment was reported after estrogen replacement [130]. Other studies report incidences of NAFLD in hormone replacement therapy of 5.3%, in pre-menopausal women of 3.5% and in post-menopausal women without treatment of 7.5% [131]. NAFLD in STH patients was reduced upon L-T4 treatment from 48.5% to 24.2% in a study including mainly (81.8%) women [132].

## 7. Conclusions

Estrogens and THs affect hepatic metabolism in both sexes but marked changes of these hormones occur more frequently in women than in men. Identifying correlations between NAFLD and specific hormonal changes is complicated because separation of the effects of the stimulating hypophyseal hormones from the action of the peripheral hormones is difficult. In addition, it is also difficult to assess the influence of THs independently of sex hormones because the prevalence of hypothyroidism in females: males is 5.4:1 [133]. Interpretation of estrogen effects is confused by the fact that E2 produced in the ovary and in the adipose tissues were shown to react differently. There is a need for further discussions about the reference range of TSH levels and about estimations of a relatively high prevalence (4.7%) of undiagnosed hypothyroidism [134].

Despite these limitations it can be concluded that different regulation patterns of hepatic metabolism were observed for THs, sex hormones and the respective stimulating hormones (Table 1). TH action is characterized by stimulation of catabolic and anabolic processes in glucose, lipid and cholesterol metabolism, whereas TSH stimulates anabolic processes in glucose and lipid metabolism and cholesterol recycling (uptake and excretion). Estrogens promote glycogen synthesis, lipid catabolism and cholesterol recycling. The role of androgen is less well defined because a U-shaped dose-dependency is suspected [102]. FSH has an anabolic function in glucose and cholesterol metabolism. These effects can explain why increased FSH levels in post-menopausal women increased gluconeogenesis and cholesterol synthesis, whereas relatively little changes are seen in men, where FSH levels are lower.

The combination of raised TSH levels and increased FSH levels in post-menopausal women may act synergistically on gluconeogenesis, cholesterol accumulation and lipogenesis. The differences in circadian levels may support an independent action because TSH levels peak in late night/early morning and FSH levels are highest in the afternoon [109,110]. The age-dependent changes in TSH and FSH levels and in NAFLD prevalence may support the hypothesis of independent and additive effects of both hormones. FSH levels in women peak between 55–70 years of age and subsequently decrease [135]. TSH levels in women were higher in the age group of 40–50 years compared with younger women [55]. Levels remained stable for about twenty years and increased again in the group of 70–90 year old women. The prevalence of NAFLD in women has been reported to increase steadily from the age of 40 years to the age of >70 years [136]. This age-dependency of NAFLD could result from a combination of increased TSH and FSH levels. L-T4 supplementation and sex hormone replacement reversed pathological changes in NAFLD in women [131,132]. Based on these findings, sex hormone replacement therapy in peri- and early post-menopause, and L-T4 supplementation in older women with STH appear suitable to prevent the development or progression of NAFLD. In fact, the combined treatment is not unusual in postmenopausal women with STH [137]. It would be interesting to know if the substitution with both hormones is more efficient in reducing hepatosteatosis than substitution with only one hormone or with TRβ-agonists. TRβ agonists like Resmetirom and other compounds represent another treatment option for NAFLD based on TH action. Results of the ongoing trials will provide more information on the efficacy of these TRβ agonists.

## Figures and Tables

**Table 1:** Effects of thyroid hormones, estrogens and androgens on hepatic glucose, lipid and cholesterol metabolism. Upregulated processes are written in bold, downregulated processes in regular font. Abbreviation: n.d., no effects reported in hepatocytes.

Hormone	Glucose Metabolism	Lipid Metabolism	Cholesterol Metabolism
Thyroid hormones	**Gluconeogenesis** **Glycogenolysis**	**Lipogenesis** **Lipolysis**	**Cholesterol synthesis, uptake, excretion**
Estrogens	Gluconeogenesis **Glycogen synthesis**	Lipogenesis **Lipolysis**	**Cholesterol uptake, excretion,** Cholesterol synthesis
Androgens	Gluconeogenesis **Glycolysis**	Lipogenesis **Lipolysis**	**Cholesterol synthesis, uptake, excretion**
Thyroid-stimulating hormone	**Gluconeogenesis**	**Lipogenesis**	Cholesterol uptake, excretion
Follicle-stimulating hormone	**Gluconeogenesis**	n.d.	**Cholesterol synthesis,** Cholesterol uptake

## Abbreviations

ABCG	ATP-Binding Cassette subfamily G
ACAT	Acyl-CoA Cholesterol Acyltransferase
ACC 1	Acetyl-CoA Carboxylase
ACD	Acyl-CoA Dehydrogenase
Akt2	Protein Kinase B
ApoB100	Apolipoprotein B100
ATGL	Adipose Triglyceride Lipase
AQ9	Aquaporin 9
BMI	Body Mass Index
CE	Cholesteryl Ester
CETP	Cholesteryl Ester Transfer Protein
ChREBP	Carbohydrate-Responsive Element-Binding Protein
CPT-1	Carnitine Palmitoyl Transferase 1
CREB	cAMP-Responsive Element Binding Protein
CYP7A1	Cholesterol-7-α-Hydroxylase
DGAT2	Diacylglycerol O-Acyltransferase 2
E2	Estradiol
ELoVL6	Elongation of Very Long Chain Fatty Acids Protein 6
ER	Estrogen Receptor
ERR	Estrogen-Related Receptor
FABP	Fatty Acid Binding Protein
FA	Fatty Acid
FFA	Free Fatty Acid
FAS	Fatty Acid Synthase
FAT	Fatty Acid Translocase (also termed CD36)
FPPS	Farnesyl Pyrophosphate Synthase
FOXO1	Factor Forkhead Box O1
FGF21	Fibroblast Growth Factor
FSH	Follicle Stimulating Hormone
fT3	Free Triiodothyronine
fT4	Free Thyroxine
GCR	Glucocorticoid Receptor
Gi	Inhibitory G protein
G6PD	Glucose-6-Phosphate Dehydrogenase
G6Pase	Glucose-6-Phosphatase
GLUT	Glucose Transporter
GnRH	Gonadotropin Releasing Hormone
GPAT	Glycerol-3-Phosphate Acyltransferase
GPER	G Protein-Coupled ER (GPR30)
GRK	G Protein-Coupled Receptor Kinase
GS	Glycogen Synthase
GSK	Glycogen Synthase Kinase
HDL	High-Density Lipoprotein
HMG-CoA	β-Hydroxy-β-Methylglutaryl-CoA
HMGCR	β-Hydroxy-β-Methylglutaryl-CoA Reductase
HNF4a	Hepatocyte Nuclear Factor 4a
IkB	Ikappa B
ICSH	Interstitial Cell Stimulating Hormone
IR	Insulin Resistance
IRS-1	Insulin Receptor Substrate 1
JNK	c-Jun N-Terminal Kinase
KAT	2-Ketoacyl-CoA-Thiolase
LAL	Lysosomal Acid Lipase
LDLR	Low-Density Lipoprotein Receptor
LH	Luteinizing Hormone
Lp	Lipophagy
LRP1	Low-Density Lipoprotein Receptor-Related Protein
LXR	Liver X Receptor
MAFLD	Metabolic Dysfunction-Associated Fatty Liver Disease
MCAD	Medium Chain Acyl-CoA Dehydrogenase
ME	Malic Enzyme
MetS	Metabolic Syndrome
mTOR	Mammalian Target of Rapamycin
mtTFA	Mitochondrial Transcription Factor A
Mp	Mitophagy
MTTP	Microsomal Triglyceride Transfer Protein
NAFLD	Non-Alcoholic Fatty Liver Disease
NASH	Non-Alcoholic Steatohepatitis
NRF1	Nuclear Respiratory Factor 1
P	Peroxisome
PFK	Phosphofructokinase
PK	Pyruvate Kinase
PKA	Protein Kinase A
PKC	Protein Kinase C
PC	Pyruvate Carboxylase
PCOS	Polycystic Ovary Syndrome
PCSK9	Subtilisin/Kexin type 9
PDK4	Pyruvate Dehydrogenase Kinase Isoform 4
PEPCK	Phosphoenolpyruvate Carboxykinase
PGC1	PPARγ-Coactivator 1α
PI3K	Phosphatidyl-Inositol-3-Kinase
POI	Premature Ovary Insufficiency
PPAR	Peroxisome Proliferator Activated Receptor
ROS	Reactive Oxygen Species
SCD	Stearoyl-CoA Desaturase
SHBG	Sex Hormone-Binding Protein
SIRT1	Sirtuin 1
SOAT2	Sterol O-Acyltransferase 2
Spot14	SPOT 14 homolog
SRB1	Scavenger Receptor Class B1
SREBP	Sterol Regulatory Element-Binding Protein
STH	Subclinical Hypothyroidism
T2DM	Type 2 Diabetes Mellitus
T3	Triiodothyronine
T4	Thyroxine
TBG	Thyroxine-Binding Globulin
TCA	Tricarboxylic Acid Cycle
THs	Thyroid Hormones
TRH	Thyrotropin Releasing Hormone
TRβ	Thyroid Hormone Receptor β
TSH	Thyroid-Stimulating Hormone
UCP2	Uncoupling Protein 2
VLDL	Very-Low-Density Lipoprotein
